# Cell-free DNA: plays an essential role in early diagnosis and immunotherapy of pancreatic cancer

**DOI:** 10.3389/fimmu.2025.1546332

**Published:** 2025-03-07

**Authors:** Yi Wen, Gengmin Zhao, Chunhua Dai

**Affiliations:** ^1^ College of Outstanding Clinician, Jiangsu University, Zhenjiang, China; ^2^ Graduate School, Heilongjiang University of Traditional Chinese Medicine, Harbin, China; ^3^ Department of Thoracic Oncology, Affiliated Hospital of Jiangsu University, Zhenjiang, China

**Keywords:** cell-free DNA, pancreatic ductal adenocarcinoma, immunotherapy, liquid biopsy, early diagnosis

## Abstract

Pancreatic cancer is renowned for its aggressive nature and dismal prognosis, with the majority of patients diagnosed at an advanced stage. The prognosis for patients with pancreatic cancer can be improved by early diagnosis and effective treatment. Circulating cell-free DNA (cfDNA) has emerged as a promising biomarker for the early diagnosis and monitoring of pancreatic cancer. This research presents a review of circulating cell-free DNA essential role in the early diagnosis and immunotherapy of pancreatic cancer. The detection methods of cfDNA, its potential as a diagnostic biomarker, and the latest research progress in cfDNA-based immunotherapy are discussed. The findings suggest that cfDNA plays a vital role in the early detection and personalised treatment of pancreatic cancer, holding great promise for improving patient outcomes.

## Introduction

**Figure 1:**
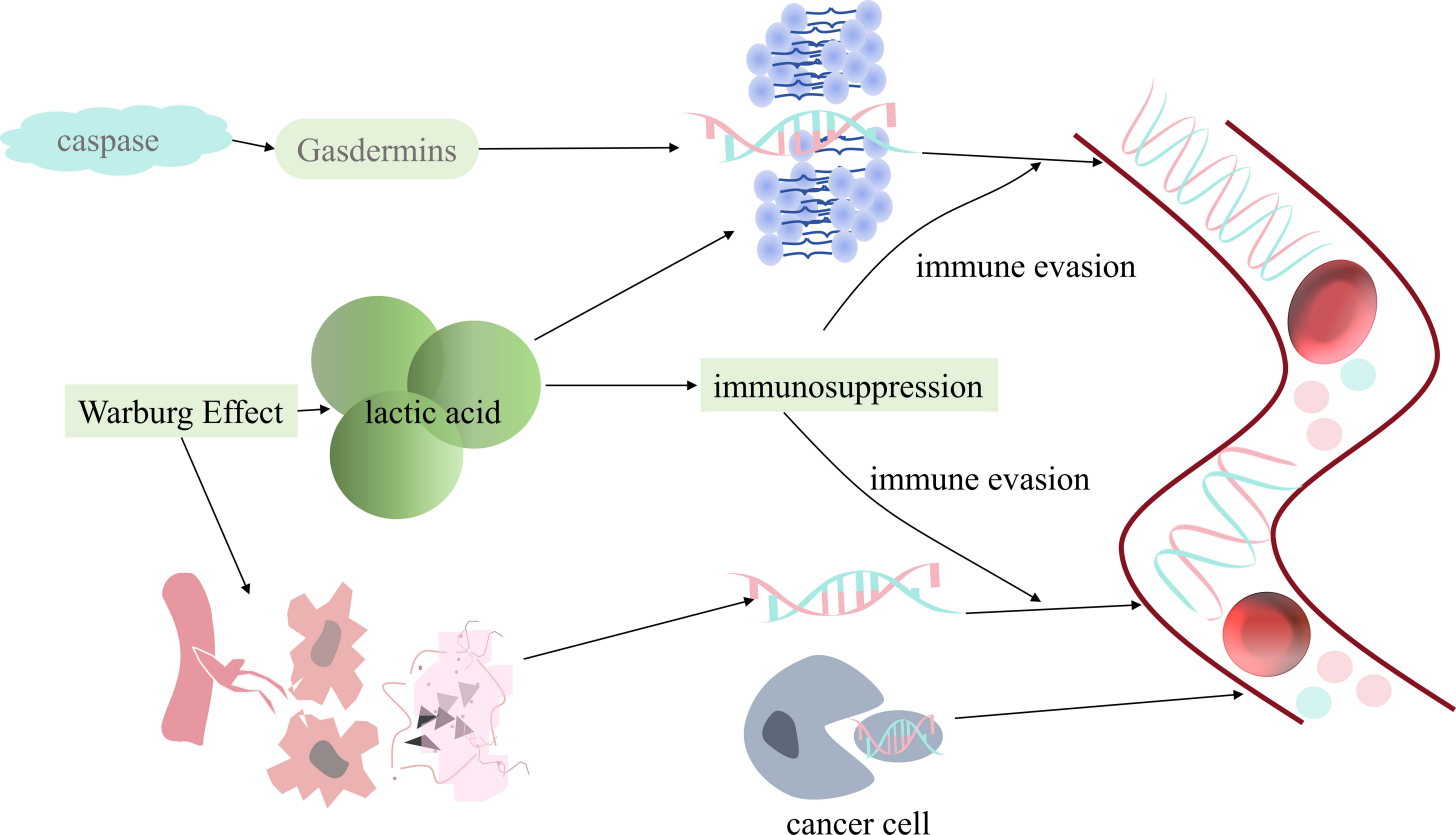
Origin of cfDNA.

Pancreatic ductal adenocarcinoma (PDAC) represents the most prevalent form of pancreatic cancer, exhibiting a high degree of aggressiveness. Despite recent advancements in diagnostic methodologies and therapeutic strategies, the 5-year survival rate for PDAC remains alarmingly low, at approximately 11% (1). PDAC is often asymptomatic in its incipient phase, with overt symptoms such as jaundice and epigastric pain becoming apparent only in the advanced stages of the malignancy. This latency in symptomatology significantly complicates the early diagnosis of pancreatic cancer. Conventional treatment modalities, including surgical resection, chemotherapy, and radiotherapy, have made some strides; however, their efficacy remains limited for the majority of patients with advanced-stage disease. In recent years, with the in-depth investigation of circulating biomarkers, particularly cell-free DNA, and the emergence of immunotherapy strategies, new hopes have been provided for improving the prognosis of patients with PDAC. cfDNA refers to short fragments of DNA found in the circulation that originate from various cell types. These DNA fragments typically range in length from 160 to 180 base pairs and are primarily categorized into cfDNA derived from normal cells, cfDNA derived from tumor cells, and fetal cfDNA (2). In pancreatic cancer, due to the highly active growth and death of tumor cells, the concentration of cfDNA in the blood of patients with pancreatic cancer is significantly higher than in normal conditions. For example, the rapid development of tumor leads to tumor ischemia, hypoxia and necrosis, or tumor tissue necrosis after radiotherapy and chemotherapy, releasing cf DNA (3). In addition, after cysteinyl aspartate specific proteinase(Caspase) is activated, the cut DNA fragments form apoptosis bodies, which escape during the immune phagocytosis process (4). Moreover, Gasdermins family proteins alter cell membrane permeability, promote cellular pyroptosis, and release cfDNA (5). In addition to this, abnormal lysosomal degradation can lead to DNA escape (6, 7). At the same time, tumor cells actively secrete multivesicular bodies that encapsulate DNA fragments by means of exosomes (8). Since pancreatic cancer is characterized by the Warburg effect as a prominent metabolic feature, the high amount of aerobic glycolytic metabolism leads to an acidic cellular microenvironment, which affects the stability and permeability of the cell membrane and leads to DNA escape (9, 10). In addition, neutrophil extracellular traps release cfDNA while trapping tumor cells (11). Moreover, aberrant glutamine metabolism leads to aberrant histone modification, which in turn leads to chromatin loosening and DNA release (12)(**Figure 1**).Therefore, analyzing cfDNA in the blood of pancreatic cancer patients is of great importance for the diagnosis, treatment, and prognosis of pancreatic cancer.

The aim of this article is to illustrate the critical role of circulating cfDNA in the early diagnosis and immunotherapy of pancreatic cancer, and to provide insight into the important value of cfDNA as a liquid biopsy marker. And we analyzed the interaction of cfDNA with the tumor immunosuppressive microenvironment in the development of pancreatic cancer, revealing the unique advantages of cfDNA in the early screening of the disease, therapeutic monitoring and prognostic assessment. Meanwhile, this paper elucidates its advantages in monitoring immunotherapy efficacy and guiding individualized treatment regimens.

## cfDNA and PDAC

Research in the early diagnosis of pancreatic cancer indicates that compared to the sole detection of the traditional pancreatic cancer tumor marker CA199, the combined detection of CA199 with cell-free miRNA (cf-miRNA) and exosomal miRNA (exo-miRNA) demonstrates higher sensitivity and specificity (13). This research is a significant advancement in the early detection of pancreatic cancer, characterized by its high detection efficiency, minimal economic burden, and the advantage of being a non-invasive testing method. Another study has demonstrated that a combined detection method based on protein and methylated circulating cell-free DNA signatures is effective in identifying patients with early-stage pancreatic cancer, significantly outperforming the use of traditional markers such as CA19-9 alone (14). This approach not only exhibits robust detection efficacy across all stages of pancreatic cancer but also demonstrates high sensitivity and specificity in the early stages. In spite of the advancements in the early detection of pancreatic cancer, clinical management of the disease continues to be challenging due to suboptimal treatment outcomes. The primary obstacles lie in the intricate tumor microenvironment of pancreatic cancer, characterized by its significant heterogeneity and a pronounced capacity for therapeutic resistance (15). These factors collectively contribute to the limited efficacy of current treatment modalities in the clinical setting. PDAC is distinguished by an abundant desmoplastic stroma, replete with fibroblasts, macrophages, and a panoply of immunosuppressive cells, which collude to establish a formidable immunosuppressive niche (16).

**Figure 2:**
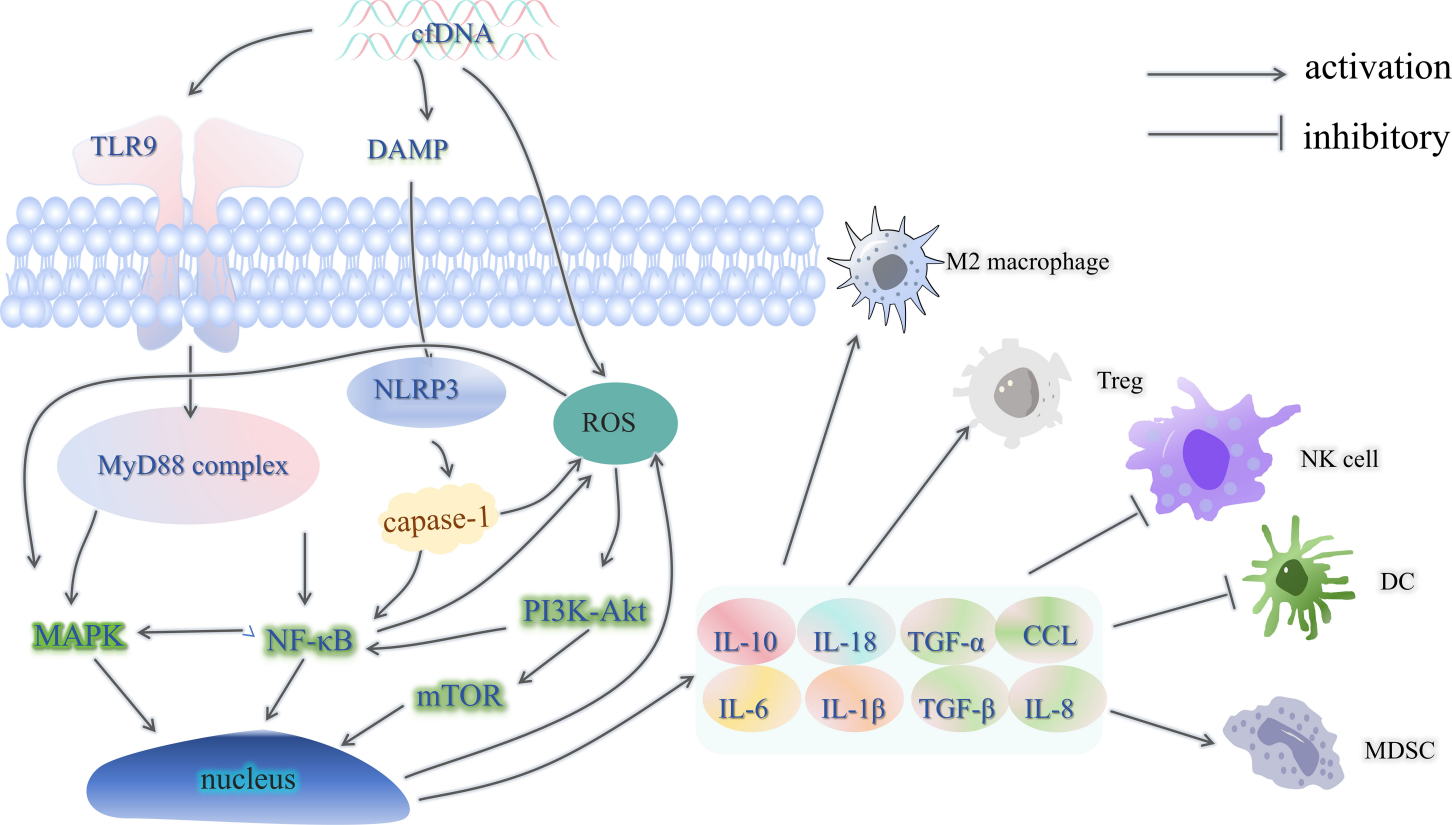
cfDNA and the immunosuppressive microenvironment. TLR9, toll-like receptor 9; MyD88, myeloiddifferentiationfactor88; DAMP, damage-associated molecular patterns; NLRP3, NOD-like receptor thermal protein domain associated protein 3; Treg, regulatory cells; Caspase, cysteinyl aspartate specific proteinase; ROS, reactive oxygen species; TNFα, tumor necrosis factor-α; IL, interleukin; CCL, chemoattractant cytokine ligand; MDSC, myeloid-derived suppressor cells; PD-1/PD-L1, programmed cell death protein-1/programmed cell death ligand-1; DC, dendritic cells.

As shown in **Figure 2**, cfDNA can bind to toll-like receptor 9 (TLR9) on the plasma membrane of immune cells, which will activate the downstream NF-κB signaling pathway, and after the formation of the NF-κB core complex, it will be transferred to the nucleus, activate the transcription of downstream genes, and stimulate the cells to produce and release cytokines and chemokines (17). The NF-κB signaling pathway also activates the MAPK signaling pathway, which regulates cell proliferation, apoptosis and inflammatory factor release (18). However, studies on the direct activation of the MAPK signaling pathway by cfDNA are still insufficient, and more in-depth mechanistic studies are needed in the future. cfDNA has been demonstrated to activate the NOD-like receptor thermal protein domain associated protein 3(NLRP3) inflammasome via the damage-associated molecular patterns (DAMPs) damage mechanism (19). This, in turn, activates caspase-1. Activated caspase-1 further activates the NF-κB signaling pathway and generates reactive oxygen species (ROS), which in turn activates the mTOR signaling pathway downstream of the PI3K-Akt signaling pathway (20). As a result, cells produce and release large amounts of inflammatory factors, chemokines, which play a role in the formation of an immunosuppressive microenvironment by enhancing immunosuppressive cell recruitment and function, promoting macrophage polarization to the M2 phenotype, inhibiting effector T-cell function, increasing the level of programmed cell death protein-1/programmed cell death ligand-1(PD-1/PD-L1) expression, and remodeling tumor stroma and fibrosis (21–23). For example, interleukin-10(IL-10) stimulates regulatory (Treg) cells to further secrete IL-10 and also induces upregulation of the immune checkpoint protein PD-L1 in monocytes, which in turn reduces CD8+ T cell infiltration in the tumor microenvironment (24). IL-18 can synergize with IL-10 to accelerate macrophage M2-type polarization, and IL-18 can directly inhibit CD8+ T cell activity (25, 26). Moreover, high expression levels of IL-6 were associated with low-density subpopulations of CD3+ and CD4+ T cells as well as FOXP3+ cells (27). Tumor necrosis factor-α(TNFα) and IL-1β are the main inducers of IL-8 expression, and IL-8 can recruit myeloid-derived suppressor cells (MDSC) and reduce the efficacy of tumor immunotherapy (28, 29).

**Figure 3:**
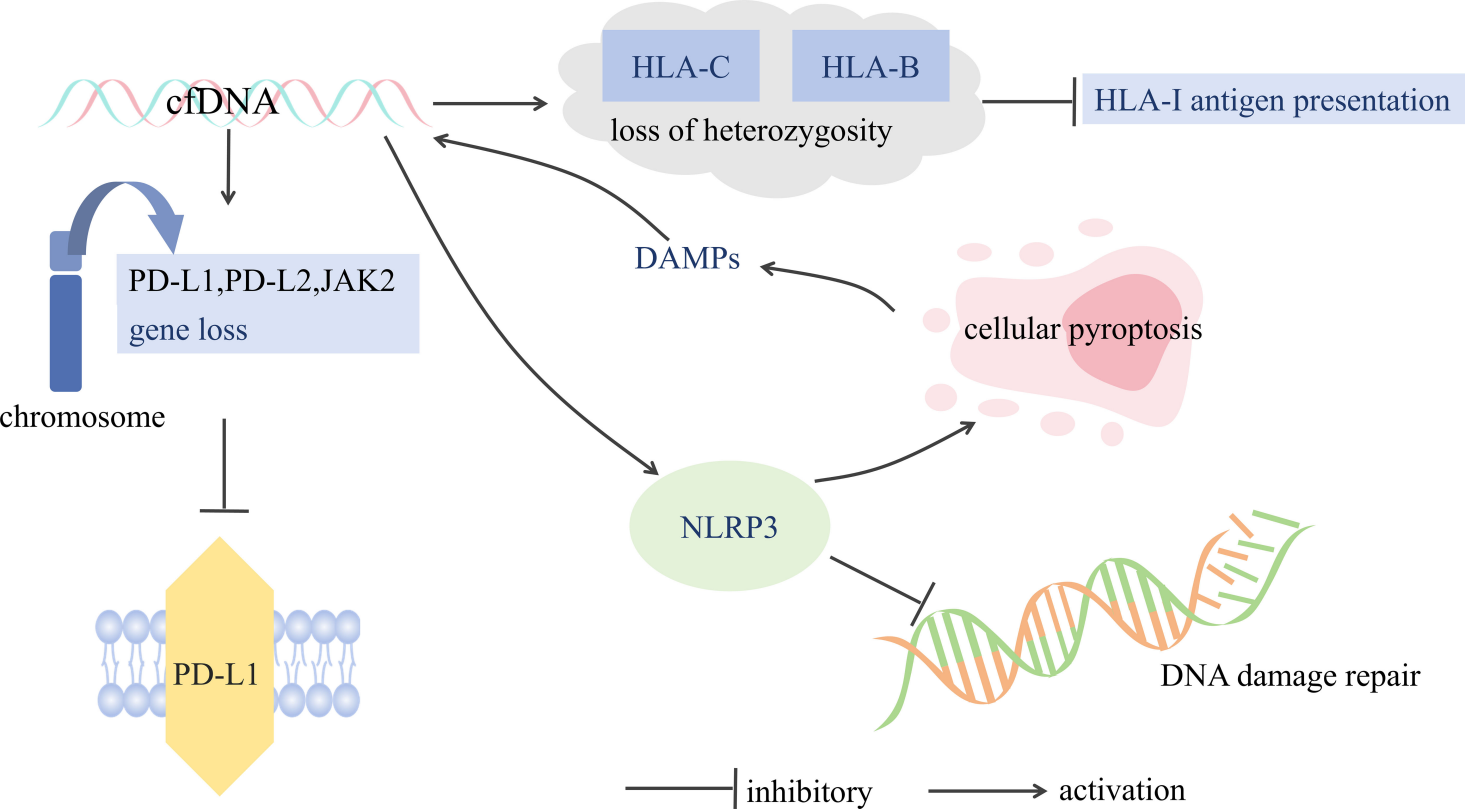
cfDNA and immune evasion.

This intricate microenvironment effectively subverts the immune surveillance and effector mechanisms, thus forestalling the development of anti-tumor immunity (**Figure 3**) (30). In addition this, Heterozygous deletions of genes in cfDNA, such as HLA-C and HLA-B, will result in a decrease in the presentation of the tumor cell surface antigen, human leukocyte antigen class I (HLA-I), decreasing immune recognition and attack by the immune system, leading to immune escape (31). In addition, cfDNA analysis detected loss of chromosome 9p, which includes the PD-L1, PD-L2 and JAK2 genes (32). The loss of these genes may lead to decreased expression of PD-L1 on the surface of the tumor cells, which reduces their ability to bind to PD-1 antibodies, evading the immune system and decreasing the efficacy of immunotherapy (33, 34). In addition, cfDNA-activated inflammatory vesicles, such as NLRP3, can lead to pyroptosis, which further releases DAMPs, which can further activate inflammatory vesicles, forming a positive feedback loop, leading to tumor cell proliferation and metastasis, and at the same time affecting the DNA damage repair mechanism of the tumor cells, thus increasing the tolerance of tumor cells to chemotherapeutic drugs and radiotherapy (20, 35, 36). In addition to this, detection of cfDNA allows for the detection of drug-resistant mutations that occur in tumor cells, such as mutations in the NTRK tyrosine kinase domain, which also lead to inactivation of the TRK inhibitor, and thus to drug resistance (37).

Moreover, PDAC manifests profound genetic and phenotypic heterogeneity, with substantial intertumoral and intratumoral variability in genetic alterations and cellular phenotypes (38). Such heterogeneity confounds the utility of uniform treatment protocols and necessitates a more nuanced approach to therapy. Even with the adoption of gemcitabine-based standard chemotherapeutic regimens, the survival benefit is transient and accompanied by significant toxicities (39–41). Consequently, the emerging field of immunotherapy, which seeks to harness the body’s own immune system to target cancer cells, holds great promise. Additionally, the utilization of circulating cell-free DNA as a non-invasive biomarker for early detection and monitoring of treatment response introduces a novel paradigm in personalized medicine (42). By integrating insights from cfDNA analysis with immunotherapeutic strategies aimed at reprogramming the tumor microenvironment, we stand to not only improve the efficacy of pancreatic cancer treatment but also reduce the burden of toxicity, thereby offering a more targeted and patient-centric approach to combat this devastating disease.

## cfDNA plays a unique role in immunotherapy for pancreatic cancer

In the context of pancreatic ductal adenocarcinoma, the high prevalence of KRAS mutations, observed in upwards of 90% of cases, is a widely recognized molecular characteristic (43). The utilization of circulating cell-free DNA analysis in peripheral blood samples has emerged as a promising method for the non-invasive detection of such oncogenic mutations. Moreover, the application of next-generation sequencing (NGS) technologies to cfDNA allows for the identification of other prevalent driver mutations, including those within TP53, CDKN2A, and SMAD4 genes (44). This liquid biopsy strategy presents a novel diagnostic tool for the initial detection, molecular stratification, and longitudinal monitoring of treatment response in patients with PDAC. It has the potential to refine personalized therapeutic approaches and enhance clinical management, thereby potentially improving patient outcomes (45). Additionally, cfDNA may harbor epigenetic modifications such as methylation markers (46, 47). The analysis of cfDNA in patients with early-stage pancreatic cancer has the potential to identify individuals who are likely to respond to immunotherapy, select the most appropriate immunotherapeutic agents, and guide combined treatment strategies. This approach holds significant importance in the precision treatment of cancer, as it enables the customization of therapeutic interventions based on the molecular profile of the patient’s tumor, thereby enhancing the efficacy of immunotherapeutic interventions.

Many studies have evaluated the efficacy of immune checkpoint inhibitors (ICIs) in solid tumors with microsatellite instability (MSI-H) by detecting the MSI-H status in tissue biopsies (48, 49). However, this approach is invasive and can be more costly. Therefore, detecting MSI-H in circulating cell-free DNA from blood offers a non-invasive alternative that can effectively assess the response of PDAC patients to immunotherapy. This method not only reduces the burden on patients but also provides a more accessible and potentially less expensive way to determine the suitability of ICIs for individual patients, thereby personalizing immunotherapy strategies (49). In addition to its role in assessing responsiveness to immunotherapy, cfDNA can also be used to evaluate the prognosis of patients after immunotherapy. A decrease in cfDNA levels is often associated with better treatment outcomes and longer survival times (50). Conversely, an abnormal increase in cfDNA levels in patients undergoing immunotherapy may indicate genuine tumor progression, preventing unnecessary changes in treatment (51, 52). Moreover, by analysing the cfDNA of pancreatic cancer, which is elevated after progression, it is possible to explore which gene fragment is mutated to make the immunosuppressant resistance leading to the progression of the disease, and whether the study of this new mutated fragment can discover new immune targets or reduce drug resistance, which is also important for the research of oncology treatment.

It has been demonstrated that personalised RNA neoantigen vaccines can elicit a robust immune response in patients with pancreatic cancer (53, 54). The vaccine-induced T cells exhibit a high degree of quality, with the capacity to persist in tumour tissue and identify and eliminate tumour micrometastases (53). Hence, in the future, the extraction of cfDNA from patients with advanced pancreatic cancer could pave the way for the development of patient-specific RNA neoantigen vaccines, offering individualized immunotherapeutic strategies for those with progressive disease. Interestingly, in a study of periodontitis, cfDNA was an important inflammatory mediator in periodontitis and was associated with systemic diseases such as rheumatoid arthritis, sepsis, atherosclerosis and cancer (55). During the acute phase of myocardial infarction, the levels of cfDNA in the blood increase, which can stimulate the activation of M1 macrophages, enhance the secretion of IL-6, and inhibit the release of IL-10 (56). Elevated cfDNA exacerbates the inflammatory response, and its pro-inflammatory effects can promote the activation of fibroblasts and collagen deposition, leading to cardiac fibrosis and impacting cardiac function (56, 57). It seems reasonable to posit that in patients with pancreatic cancer, cfDNA may also exert comparable pro-inflammatory effects. High concentrations of circulating free DNA have been observed in the vicinity of pancreatic tumours. This cfDNA may contribute to the development of stromal components within the tumour through a number of mechanisms, including the promotion of intratumoural fibrosis and the creation of a hypoxic microenvironment. It is hypothesised that in the context of early-stage pancreatic cancer, the elevated levels of circulating free DNA in the vicinity of the tumour may function as a tumour antigen, potentially recognisable by T cells. Nevertheless, as the tumor progresses, it is conceivable that upon binding to T cells, this cfDNA may inhibit the immune response and result in T cell apoptosis. This change from promoting immune function to suppressing tumor immunity requires multifrequency follow-up and monitoring. If cfDNA tends to increase during patient follow-up, this may indicate a change in tumor status, where the immune component is “rebelling” and previous treatments may have become resistant, requiring other, more sensitive, drug-resistant regimens.The choice of treatment regimen after resistance can be made by elevated cfDNA, because the gene fragments responsible for the “ rebelling “ may be hidden in the serum cfDNA, which is a better test than biopsy of the patient’s tissues again. And this needs to be further confirmed in clinical cohort studies.

## Challenges of cfDNA in immunotherapy

**Figure 4:**
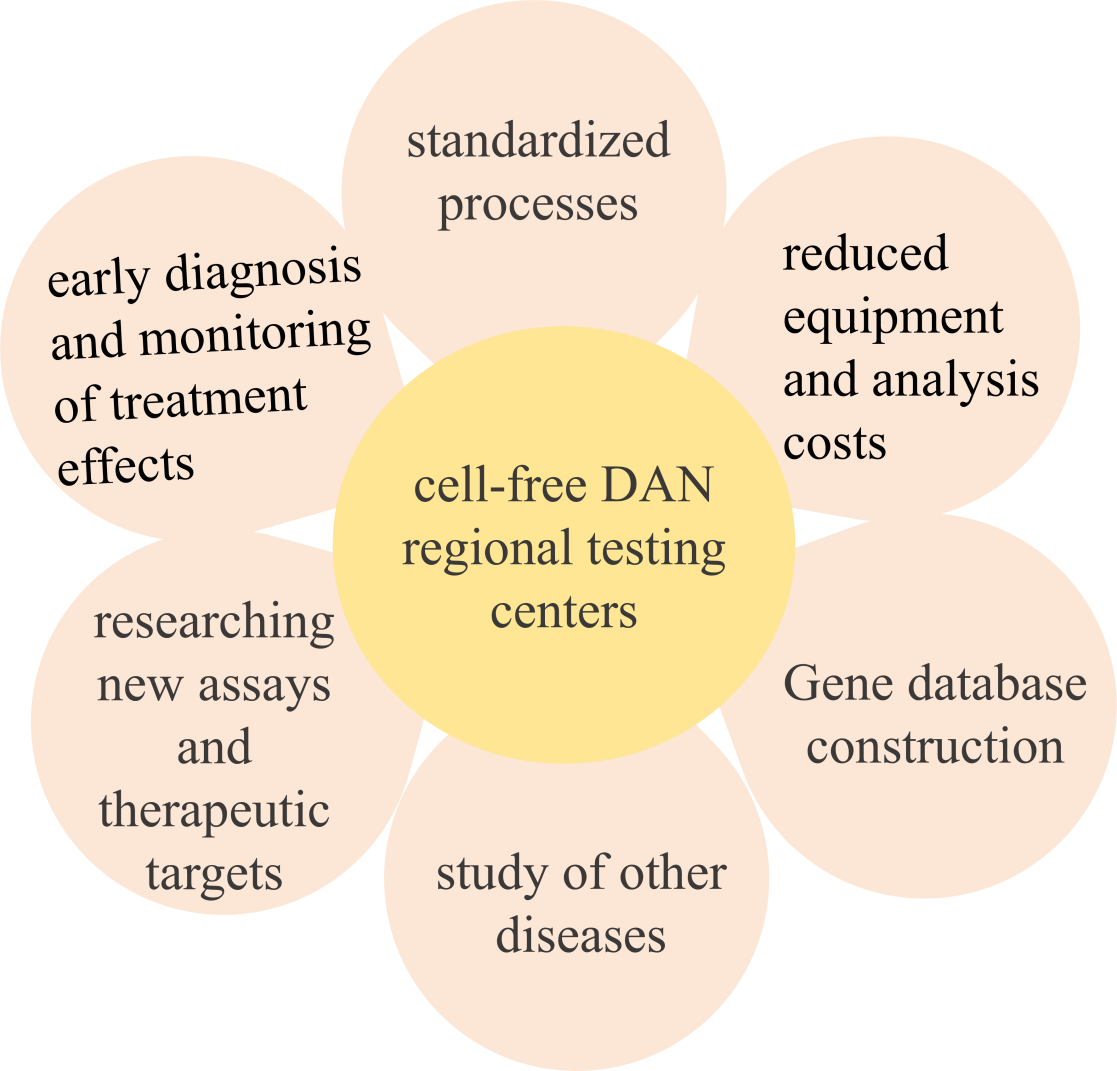
Significance of building regional centers.

Due to the relatively low concentration of cfDNA in blood, especially in patients with low tumour loads, this may lead to insufficient sensitivity of the assay, while the low concentration of cfDNA also poses a challenge to analytical techniques. Gene sequencing, and more sensitive techniques and methods are needed to extract and analyse cfDNA. There is also the challenge of not having a standardized process for specimen acquisition, storage and testing. Developing and researching new detection and analytical techniques, this represents a significant financial burden to the clinic hospitals. We believe that regional centres for cfDNA testing could be set up in laboratories where they are available, and that hospitals would only need to provide specimens, which would be preserved and transported to the regional centre, which would reduce the burden on each hospital. There are already research techniques for detecting cfDNA in experiments, such as cfTAPS technology for whole genome methylation sequencing of cfDNA. cfTAPS technology is capable of generating high-quality genome methylation data from a small amount of cfDNA (10 ng), which allows the identification of the tissue origin of the cfDNA, and is promising for applications (58). However, the technology has the problems of insufficient experimental sample size and insufficient database information, which need to further expand the research samples and carry out multi-centre studies in hospitals in different regions. The economic cost of the technology is also too high, and the establishment of a regional centre for cfDNA testing would be a perfect solution to the above problems. Therefore, establishing regional centers for cfDNA detection could have the following advantages:

1. Could significantly contribute to the early detection of pancreatic cancer in patients.
2. Could reduce the equipment costs for each hospital.
3. It is of the utmost importance to implement consistent testing protocols and quality control criteria in order to enhance the reliability and comparability of cfDNA testing. And Regional testing centers can use the same set of processes.
4. Regional testing centers can also reduce per-patient testing and analysis costs by testing specimens from multiple hospitals at once. Alternatively, highly specific sites in cfDNA can be detected, it’s also a way to reduce the cost per patient.
5. The establishment of regional centers for cfDNA testing can facilitate more in-depth research in the field, such as exploring the application of cfTAPS technology in detecting other genetic and epigenetic information in cfDNA, utilizing cfTAPS technology to detect and develop new cfDNA biomarkers for various cancer types, using cfTAPS technology to study the tumor microenvironment and host response, providing new targets and strategies for drug development. Additionally, it can explore the application of cfTAPS technology in the diagnosis and treatment monitoring of other diseases, such as hereditary diseases, autoimmune diseases, and infectious diseases. Therefore, we believe that establishing cfDNA testing regional centers in well-equipped laboratories is an excellent initiative (**Figure 4**).

Clonal Hematopoiesis of Indeterminate Potential (CHIP) is indeed an important issue in cfDNA testing. CHIP is a common genetic variation in the elderly, characterized by the presence of one or more clonal hematopoietic cell populations in the blood that carry specific genetic mutations (59). In addition this, CHIP-associated mutations usually occur in genes associated with tumourigenesis, such as TP53, KRAS, NRAS, and PIK3CA (60, 61). CHIP-associated mutations release cfDNA into the bloodstream, which may have similar mutations to tumour-derived cfDNA, potentially leading to false-positive results in cfDNA testing and reducing the specificity of cfDNA testing, which can confound cfDNA test results and lead to misdiagnosis or missed diagnosis. It is therefore necessary, in practice, to reduce CHIP interference and improve the accuracy of cfDNA detection. CHIP can be identified and detected through the use of high-throughput sequencing, single-cell sequencing, and microarray technology (62). Mutational Heterogeneity and Outlier Discovery Tool 2 (Mutect2) has been found to be able to detect single nucleotide variation and insertion/deletion variation, with preliminary filtering based on sequencing depth, variant allele frequency, base quality, and other factors to improve the accuracy of detection, and is an effective tool for identifying and classifying CHIP variants (63).Alternatively, it can be identified by analysing the clonality of the mutation, as CHIP-associated mutations are usually monoclonal, whereas tumour-associated mutations are usually polyclonal (60, 64). In addition to this, attention needs to be paid to the patient’s clinicopathological features, such as the patient’s age, smoking history, and chronic inflammatory diseases, which are considered in the context of the patient’s overall situation (65).

Following an exhaustive examination of the subject matter, it can be stated that cfDNA is of paramount importance in the context of pancreatic cancer immunotherapy. As a non-invasive biomarker, circulating free DNA can effectively reflect the tumour load, genomic alterations and response to immunotherapy in patients with pancreatic cancer. cfDNA analysis can assist in the identification of immunotherapy responders and non-responders, and in the development of a personalised treatment plan for each patient, with the aim of achieving precision immunotherapy. cfDNA can be employed as a dynamic monitoring tool to assess the effectiveness of immunotherapy. Once the desired effect of immunotherapy has been achieved, cfDNA testing can be employed to monitor minimal residual disease, assess the risk of disease recurrence, and inform subsequent treatment decisions. Once a tumour has developed resistance to immunosuppressants, analysis of circulating tumour DNA can facilitate the identification of the underlying mechanisms of immunotherapy resistance, thereby providing insights that may inform strategies for overcoming this resistance. It is therefore anticipated that a comprehensive investigation into the function of cfDNA in pancreatic cancer immunotherapy will yield novel insights and methodologies for the early diagnosis, efficacy assessment and prognosis prediction of pancreatic cancer.
